# A comprehensive risk assessment method for hot work in underground mines based on G1-EWM and unascertained measure theory

**DOI:** 10.1038/s41598-024-56230-y

**Published:** 2024-03-13

**Authors:** Xiaoqiang Ding, Xiangliang Tian, Jinhui Wang

**Affiliations:** 1 College of Ocean Science and Engineering, Shanghai Maritime University, Shanghai, 201306 China; 2 Institute of Mine Safety Technology, China Academy of Safety Science and Technology, Beijing, 100012 China

**Keywords:** Hot work, Order relationship method (G1), Entropy weight method (EWM), Combined weighting, Unascertained measurement theory, Electrical and electronic engineering, Mineralogy, Energy security

## Abstract

A risk assessment method for hot work based on G1-EWM and unascertained measurement theory was proposed to prevent hot work accidents in underground mines. Firstly, based on the risk influencing factors and classification criteria for underground hot work operations in mines, a single indicator measurement matrix was constructed using unascertained measurement theory; Secondly, a risk assessment index system for mine underground hot work operations was established. The combination weight coefficient of each index was determined using the order relationship analysis method (G1) and entropy weight method (EWM) and coupled with the single index measurement evaluation vector to calculate the multi-index comprehensive evaluation vector of the evaluation object; Finally, the model was validated and examined using engineering examples, and the evaluation level was determined using confidence identification criteria. The results showed that the proposed method, when used to evaluate the risk of hot work operations in tunnels and vertical shafts in metal mines, produces risk levels that are in line with reality III (Moderate Risk) for the vertical shaft and IV (High Risk) for the tunnels. The evaluation model results are consistent with the risk evaluation results the whole process of on-site hot work, which verifies the model feasibility. A unique strategy and method for risk management in hot work operations in underground mines is provided by the combination of weighting and unascertained measure models, which has theoretical and practical value. Future research could focus on refineing this model by exploring the applicability in diverse mining environments and integrating advanced analytical techniques to enhance the predictive accuracy and operational efficiency.

## Introduction

Hot works are an integral part of the construction, operation, routine maintenance, and inspection processes in underground mines. In recent years, mine fire accidents have occurred frequently, such as the underground fire accident in the Liaoning Xingli Mine in December 2015, the mine fire accident in the Hongxing Iron and Steel Mine in August 2016, and the two-mine fire and explosion accidents in Yantai in 2021^1–3^. These fire accidents were caused by unauthorized hot work operations igniting nearby combustible materials, which generated a large amount of smoke and toxic and harmful gases spreading downwind along the tunnel, causing secondary hazards such as poisoning and asphyxiation of underground personnel, and resulting in serious casualties and economic losses. Therefore, it is of great significance to reasonably analyze the risk factors involved in the whole hot work process in mines and perform systematic and effective risk evaluation studies to reduce and control the risk of hot work accidents.

A hot work permit is a prerequisite for hot work operations, and many scholars have researched the approval management and system of hot work operations^4,5^. However, investigations have shown that in recent years, hot work accidents have occurred even with the issuance of hot work permits, and these studies have exerted a negligible impact on preventing hot work accidents. In the risk assessment of hot work, the purpose of risk assessment is to provide evidence-based information and analysis to make informed decisions on how to treat particular risks and how to select between options^6^. He et al.^7^ used AHP and fuzzy comprehensive evaluation methods to evaluate the risk of oil and gas pipeline hot work operations and utilized the questionnaires and AHP to determine the weight coefficients of each index to obtain the hazard level of the evaluation object. Mousa et al.^8^ assessed the risks of fire, explosion, and toxic gas release during hot work operations in sulfur-containing natural gas pipelines using the fuzzy analytic hierarchy Process (FAHP). They found that the condition of the natural gas pipelines exerted a significant impact on the final risk level. Dong et al.^9^ analyzed the explosion incident during hot work inside a benzene storage tank at the Shanghai Saikao Chemical Plant in 2018 using FLACS software and a multilayer bow-tie model. They concluded that the primary cause of the accident was the lack of sustained flammable gas detection during the hot work process. Wang^10^ quantitatively analysed the unsafe behaviours of personnel during hot work operations using Bayesian networks. They identified that the main causes of hot work accidents include the absence of a hot work permit, failure to isolate the production system, lack of risk assessment, failure to remove combustibles, and inadequate fire prevention measures. In the field of statistics and analysis of hot work accidents, Shin and Kariuki^11,12^ noted that the proportion of human error in the causes of hot work accidents is significantly higher than the probability of equipment failures and operational approval errors. Xu et al.^13^, through text mining and deep learning, analyzed 267 cases of unstructured text data related to hot work accidents. They automatically extracted and predicted accident causes by category and concluded that the primary causes of hot work accidents are the lack of flammable gas detection or continuous monitoring, incomplete combustible material clearance, and inadequate protective measures. The aforementioned models have certain advantages in dealing with hot work operations in chemical industrial parks and natural gas pipeline hot work. However, the underground mining environment is complex with numerous influencing factors^14^. When determining the weight of risk factors for underground hot work operations, it is challenging to avoid the impact of subjective decision-making. Additionally, some methods suffer from calculation redundancy and low practicality, making them difficult to apply to risk assessment in mining hot work operations. Therefore, it is imperative to explore a model that can apply to both pre-hot work safety management in mining and serve as a hazard assessment model during the hot work construction process.

Many researchers have carried out a lot of risk assessment work in underground mining^15^, vehicle transportation^16,17^, belt conveyor^18^ and other accidents, and have systematically summarized many models and methods suitable for underground mine risk assessment^19,20^, such as analytic hierarchy process(AHP) and fault tree analysis(FTA), support vector machine(SVM), TOPSIS and neural network methods. However, there has been relatively less research on hot work operations in underground mining, and traditional risk assessment methods have limited practicality in addressing ambiguity, complexity, and uncertainty. The unascertained mathematical theory was proposed by Wang Guangyuan in 1990^21^. Different from fuzzy information and gray information, the theory can judge the real status of affairs when people have insufficient information, thereby providing decision-makers with the best decision-making assistance. On the one hand, it can quantitatively and effectively analyze various uncertain factors that exist in the process of mine fire operations. On the other hand, it can avoid incomplete evaluation targets caused by the uncertainty of influencing factors. And it is not known that the measurement theory model has been widely used in risk assessment of geotechnical engineering, construction sites, and subway workplaces.

Considering the above, the present study aims to identify the risk factors that exist in the entire process of underground hot work operations in mines, ranging from approval to completion. A novel hot work risk assessment model is established that incorporates a combination weighting approach and the unascertained measure theory. This model combines weighting methods through the order relationship method (G1) and entropy weighting method (EWM) to calculate the weighting coefficients of each indicator, and combines the single-indicator measurement function of each evaluation indicator to calculate a comprehensive vector of measurements and determine the risk level of the evaluation object^22–24^. Furthermore, the model is applied in the practical risk assessment of hot work operations in mines to verify its feasibility and accuracy, providing an effective analytical method for mine hot work management.

## The unascertained measure theory risk evaluation model

Assume that the hot work risk evaluation object X corresponds to n evaluation indices, expressed as $X=\left\{{x}_{1}, {x}_{2}, {\ldots , x}_{n}\right\}$. If $x_{i} \;i = 1,{ }2, \ldots ,{\text{ n}}$ has *m* risk levels, represented by $R_{k} k = 1,{ }2,{ } \ldots ,{\text{ m}}$, then it can form an *m*-dimensional evaluation vector ${X}_{i}=\left\{{x}_{i1}, {x}_{i2}, {\ldots , x}_{im}\right\}$, where ${x}_{ij}$ denotes the *j*th risk level of evaluation indicator ${x}_{i}$ in the hot work operation process.

By dividing ${x}_{ij}$ into P risk levels, the evaluation space $T=\left\{{C}_{1}, {C}_{2}, {\ldots , C}_{p}\right\}$ of ${x}_{ij}$ can be obtained. If the risk level of *k*th is higher than the (*k* + 1)th risk level, this is denoted as ${C}_{k}>{C}_{k+1}$, moreover ${C}_{1}>{C}_{2}>{\ldots >C}_{p}$, and $\left\{{C}_{1}, {C}_{2}, {\ldots , C}_{p}\right\}$ is denoted as an ordered partition class^25^.

### Determination of the single-indicator unascertained measurement matrix

According to relevant information of the evaluation object and the definition of an unascertained measure used to establish the single-indicator measure distribution function for whole-process risk evaluation of hot work operations, the single-indicator measure matrix of the evaluation object can be expressed as Eq. (1)^26–29^:

1 $$\begin{array}{c}{\left({\upmu }_{ijk}\right)}_{m\times p}=\left[\begin{array}{cccc}{\mu }_{i11}& {\mu }_{i12}& \ldots & {\mu }_{i1p}\\ {\mu }_{i21}& {\mu }_{i22}& \ldots & {\mu }_{i2p}\\ \vdots & \vdots & \ddots & \vdots \\ {\mu }_{im1}& {\mu }_{im2}& \ldots & {\mu }_{imp}\end{array}\right]\end{array}$$

where ${\upmu }_{ijk}$ is the degree of indicator assignment ${x}_{ij}$ at the kth risk level ${C}_{k}$.

### Determination of the weights of the evaluation indicators

#### Entropy weighting method for calculating objective weights (EWM)

The entropy weight method is based on the degree of variation in each evaluation index, and can be used to calculate the corresponding entropy weight, for obtaining the objective weight of each index^29–33^. In the whole hot work operation process, each evaluation index exhibits quantitative outline and quantitative value differences, and it is necessary to first standardize the data. The objective weight of each evaluation index can be calculated as follows:

##### Normalization of the indicators

Standardize the single-index measurement matrix. Let ${R}_{ij}$ represent the normalized indicator value. Then, the following can be obtained:

2 $$\begin{array}{c}{R}_{ij}=\frac{{x}_{ij}-{\text{min}}\left({x}_{ij}\right)}{{\text{max}}\left({x}_{ij}\right)-{\text{min}}\left({x}_{ij}\right)}\end{array}$$

##### Calculation of the information entropy of the indicators

3 $$\begin{array}{c}{e}_{j}=-\frac{1}{\mathit{ln}n}\sum_{j=1}^{n}{a}_{ij}\mathit{ln}{a}_{ij} \,\,\,\,\,\,\,\,\,j=1, 2, 3, \dots , n\end{array}$$

In the above equation, ${a}_{ij}$ is the weight of the jth indicator under the ith evaluation criterion, so ${a}_{ij}$ can be expressed as:

4 $$\begin{array}{c}{a}_{ij}=\frac{{r}_{ij}}{\sum_{j=1}^{n}{r}_{ij}}\end{array}$$

##### Determination of the objective weights for indicators

The weight of the jth indicator is:

5 $$\begin{array}{c}{v}_{j}=\frac{1-{e}_{j}}{m-\sum {e}_{j}}\end{array}$$

#### Order relationship method for calculating subjective weights (G1)

The order relationship method (G1) is a subjective weighting method proposed by Guo Yajun et al.^34,35^. This method builts upon the analytic hierarchy process (AHP) and involves ranking evaluation indicators based on their importance. Subsequently, weight can be quantitatively calculated by comparing the relative importance of adjacent indicators^36–39^. The specific steps are as follows:

##### Determination of the order relationship between the evaluation indicators

Among the risk assessment indicators $\left\{{x}_{1} , {x}_{2}, \ldots ,{ x}_{n} \right\}$ at the same level, the most important indicator, denoted as ${x}_{1}^{*}$, is selected by the expert according to the hierarchical evaluation criterion. The second most important indicator, denoted as ${x}_{2}^{*}$, is selected among the remaining n−1 indicators, and the process is continued until the last indicator factor is selected after n−1 selections, denoted as ${x}_{n}^{*}$. Theorder of the available evaluation indicator is as follows:

6 $$\begin{array}{c}{x}_{1}^{*}{>x}_{2}^{*}>{x}_{3}^{*}\ldots >{x}_{n-1}^{*}>{x}_{n}^{*}\end{array}$$

##### Calculation of the relative importance of the indicators

Referring to Table 1, which provides relative importance values for the risk assessment factors, the importance ratios ${r}_{k}$ between adjacent evaluation indicators ${x}_{k-1}^{*}$ and ${x}_{k}^{*}$ are as follows:

7 $$\begin{array}{c}{r}_{k}=\frac{{w}_{k-1}}{{w}_{k}}\end{array}$$

where ${w}_{{\text{k}}-1}$ and ${w}_{{\text{k}}}$ are the weighting coefficients of evaluation indicators ${x}_{{\text{k}}-1}$ and ${x}_{{\text{k}}}$, respectively.

**Table 1:** Assignment reference table.

Assignment of ${r}_{k}$	Instructions
1.0	Indicator ${x}_{k-1}^{*}$ and ${x}_{k}^{*}$ are equally important
1.2	Indicator ${x}_{k-1}^{*}$ is slightly more important than ${x}_{k}^{*}$
1.4	Indicator ${x}_{k-1}^{*}$ is significantly more important than ${x}_{k}^{*}$
1.6	Indicator ${x}_{k-1}^{*}$ is strongly more important than ${x}_{k}^{*}$
1.8	Indicator ${x}_{k-1}^{*}$ is extremely more important than ${x}_{k}^{*}$

##### Determination of the subjective weights of the indicators

Based on the relative importance of the indicators, the weight of the kth indicator can be calculate as follows:

8 $$\begin{array}{c}{w}_{k}={\left(1+\sum_{k=2}^{n}\prod_{i=k}^{n}{r}_{i}\right)}^{-1}\end{array}$$

The weights of the other indicators at each level are:

9 $$\begin{array}{c}{w}_{k-1}={r}_{k}{w}_{k}\end{array}$$

#### Combination weights

According to the aforementioned methods, the evaluation process of the entropy weight method heavily relies on objective data from hot work sites, while the order relationship method more notably depends on the knowledge level and subjective experience of experts. To avoid subjectivity due to subjective weighting and considering the mathematical nature of objective weighting, the order relationship method and entropy weight method are combined in this paper to calculate weights^39–41^. The combination weight ${Z}_{i}$ for each evaluation indicator can be calculated as follows:

10 $$\begin{array}{c}{Z}_{i}=\frac{{w}_{i}{v}_{i}}{\sum_{i=1}^{n}{w}_{i}{v}_{i}}\end{array}$$

where ${w}_{i}$ denotes the subjective weights; and ${v}_{i}$ denotes the objective weights.

### Determination of the multiple-indicator unascertained measurement matrix

Based on Eq. (10), the weight of the risk evaluation index of the whole hot work operation process is ${Z}_{i}$. If ${\upmu }_{ik}$ satisfies $0{\le\upmu }_{ik}\le 1$, ${\upmu }_{ik}$ can be expressed as follows:

11 $$\begin{array}{c}{\upmu }_{ik}=\sum_{j=1}^{n}{Z}_{i}{\upmu }_{ijk}\end{array}$$

The the evaluation matrix ${\left({\upmu }_{ik}\right)}_{m\times p}$ for the whole hot work operation process with multiple indicators of unascertained measurement can be obtained as:

12 $$\begin{array}{c}{\left({\upmu }_{ik}\right)}_{m\times p}=\left[\begin{array}{cccc}{\mu }_{11}& {\mu }_{12}& \ldots & {\mu }_{1p}\\ {\mu }_{21}& {\mu }_{22}& \ldots & {\mu }_{2p}\\ \vdots & \vdots & \ddots & \vdots \\ {\mu }_{m1}& {\mu }_{m2}& \ldots & {\mu }_{mp}\end{array}\right]\end{array}$$

### Identification of the confidence criterion

To comprehensively assess the risk evaluation level of hot work operations and ensure the accuracy of the assessment results, a confidence criterion is introduced. Let the confidence degree be noted as $\lambda \ge 0.5$(usually 0.6 or 0.7). If the evaluation vector is an ordered partition class and ${C}_{1}>{C}_{2}>{\ldots >C}_{p}$ , the following is satisfied:

13 $$\begin{array}{*{20}c} {P_{0} = min\left\{ {P:\mathop \sum \limits_{k = 1}^{P} {\upmu }_{ik} \ge \lambda ,\;i = 1,2, \ldots ,n} \right\}} \\ \end{array}$$

then the risk level of the evaluation object belongs to ${P}_{0}$.

## Hot work risk evaluation indicator system

### Selection of evaluation indicators

Although regulatory authorities have issued regulations to standardize hot work operations in underground mines, mining enterprises are still struggling to identify the risk factors involved in the entire hot work operation process s. Moreover, they face difficulties in defining safety measures and inspections before hot work operations and ensuring clear responsibility control throughout the hot work process. To address these issues, the factors that affect the safety of hot work operations are comprehensively analysed in this pape. The analysis considers recent hot work accidents, relevant laws, regulations, and the literature, and focuses on four key aspects: human, equipment, environment, and management factors. A total of 19 influencing factors are identified as evaluation indicators, and a risk indicator assessment system for hot work accidents is established. This system can help mining enterprises improve their safety measures and inspections and ensure the safety of hot work operations.

### Criteria for grading and quantification of evaluation indicators

Based on the characteristics of hot work operations, the evaluation space T is divided into five levels $\left\{{C}_{1}, {C}_{2}, {C}_{3}, {C}_{4}, {C}_{5}\right\}$, i.e., I, II, III, IV and V, representing an extremely low risk (I), low risk (II), moderate risk (III), high risk (IV), and extremely high risk (V), respectively. The specific grading criteria and assigned values forthe risk-influencing factors are provided in Table 2.

**Table 2:** Risk indicator classification criteria for the hot work operation.

Primary indicators	Secondary indicators	Risk classification and assignment of values				
		Class I	Class II	Class III	Class IV	Class V
Human factors	Pre-job safety training (X_1_)	Reasonable	More reasonable	General	Unreasonable	Highly unreasonable
	PPE usage (X_2_)	Perfect	Irregularities	Incomplete	More incomplete	Not wearing
	Proper operation (X_3_)	Reasonable	More reasonable	General	Unreasonable	Highly unreasonable
	Licensed operation(X_4_)	Licensed and number ≥ 3	Licensed and number ≥ 2	Licensed and number ≥ 1	Licensed but not valid	Unlicensed
Equipment factors	Hot work caution line (X_5_)	8–10 m	6–8m	4–6 m	2–4 m	< 2 m
	Safety distances for gas cylinders (X_6_)	≥ 5 m	4–5 m	3–4 m	2–3 m	< 2 m
	Cylinder stabilization (X_7_)	High stability	Strong stability	Moderate stability	Limited stability	No stability
	Grounding of welding machine shell (X_8_)	Adequate grounding	Partial grounding	Limited grounding	Minimal grounding	No grounding
	Flame-resistant cable (X_9_)	Completely flame resistant	Highly flame resistant	Moderately flame resistant	Limited flame resistance	Not flame resistant
Environmental factors	Toxic and hazardous gas detection (X_10_)	Reasonable	More reasonable	General	Unreasonable	Highly unreasonable
	Worksite combustibles (X_11_)	Safety distance > 10 m	Safety distance 8–10 m	Safety distance 5–8 m	Safety distance site 2–5 m	Safety distance < 2 m
	Worksite lighting(X_12_)	Good lighting	Better lighting	General lighting	Poor lighting	No lighting
	Pumice on the work site slab (X_13_)	No pumice	Low pumice presence	Moderate pumice presence	High pumice presence	Excessive pumice presence
Management factors	Hot work operations classification and procedures (X_14_)	Expository	Preferably	General	Relatively chaotic	Indefinite
	Hot work permit (X_15_)	Standardize	Relatively standardize	General	Relatively poor	Poor
	Risk factor identification (X_16_)	Comprehensive	Relatively comprehensive	General	Relatively poor	Poor
	Specialized emergency protocols (X_17_)	Match	Relatively matched	General	Relatively poor	Poor
	Fire extinguisher (X18)	Comprehensive	Comprehensive but substandard	Missing 1–2 pieces	Missing 3–4 pieces	None
	Warning sign (X19)	Good location and layout	Reasonable location and poor layout	Poor location and reasonable layout	Poor location and confusing layout	No visible warning signs
Assignment of qualitative indicators		5	4	3	2	1

### Implementation steps

**Figure 1:**
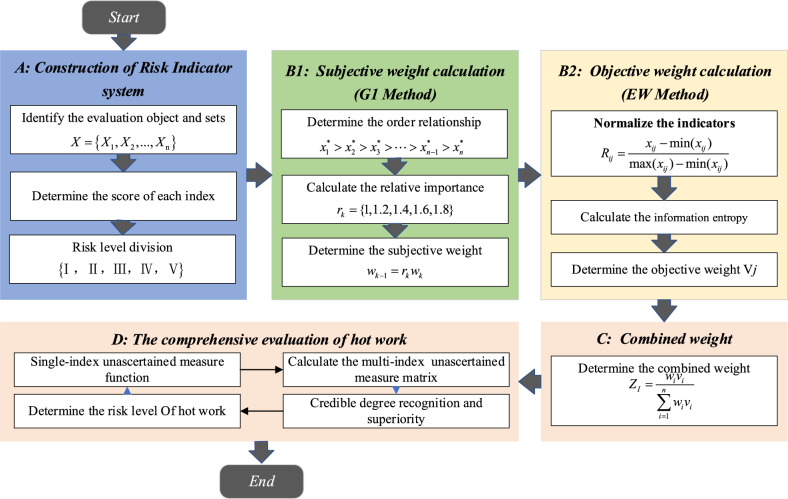
Specific steps of evaluation model for hot work operations.

Based on unascertained measure theory and G1-EWM method, a risk evaluation model for mine hot work operations is constructed. The specific steps are shown in Fig. 1.

- *Step 1* Construct a risk indicator system based on the hot work evaluation object and set the risk level for each index.
- *Step 2* Determine the subjective weights of each evaluation index using the order relationship method (G1).
- *Step 3* Determine the objective weights of each evaluation index using the entropy weight method (EWM).
- *Step 4* Calculate the combined weight of each evaluation index.
- *Step 5* Construct the single-index unascertained measurement function for the indicators according to the definition of unascertained measurement theory.
- *Step 6* Determine the multi-index unascertained measurement function for indicators.
- *Step 7* Determine the risk level of the evaluation object based on the confidence identification criteria.

## Model application

Adopting a metal mine in Yantai, Shandong Province, as an example, the underground mining operation exhibits a production scale of 330,000 t/a. The mine has been excavated down to the mid-level of − 390 m, and the area from − 390 to − 630 m is part of a new system. The construction involves six major systems: underground drainage system, a ventilation system, a water supply and firefighting system, a lifting and transportation system, a power supply and distribution system, and safety precautions. The mine also manages the beneficiation plant and production mine jointly, involving a significant number of hot work projects such as pipeline welding and equipment maintenance.

Currently, 80 personnel are qualified in terms of melting welding and thermal cutting operations. Through the analysis of various aspects, including the mine hot work operation management system, approval processes, safety training before hot work initiation, safety inspections, and personnel violations during hot work, a total of 19 risk factors related to the entire hot work operations process were selected for measurement and evaluation. Because of the uncertainty in the underground hot work operation locations, it is necessary to evaluate the risk factors for hot work operation at different locations in the underground minesto determine the corresponding level of safety protection according to the evaluation results and ensure the safety of the hot work operation process. In this paper, we evaluate the risk consideringtwo typical hot work operation scenarios: mine shafts and tunnels.

### Constructing single-indicator measurement functions

**Figure 2:**
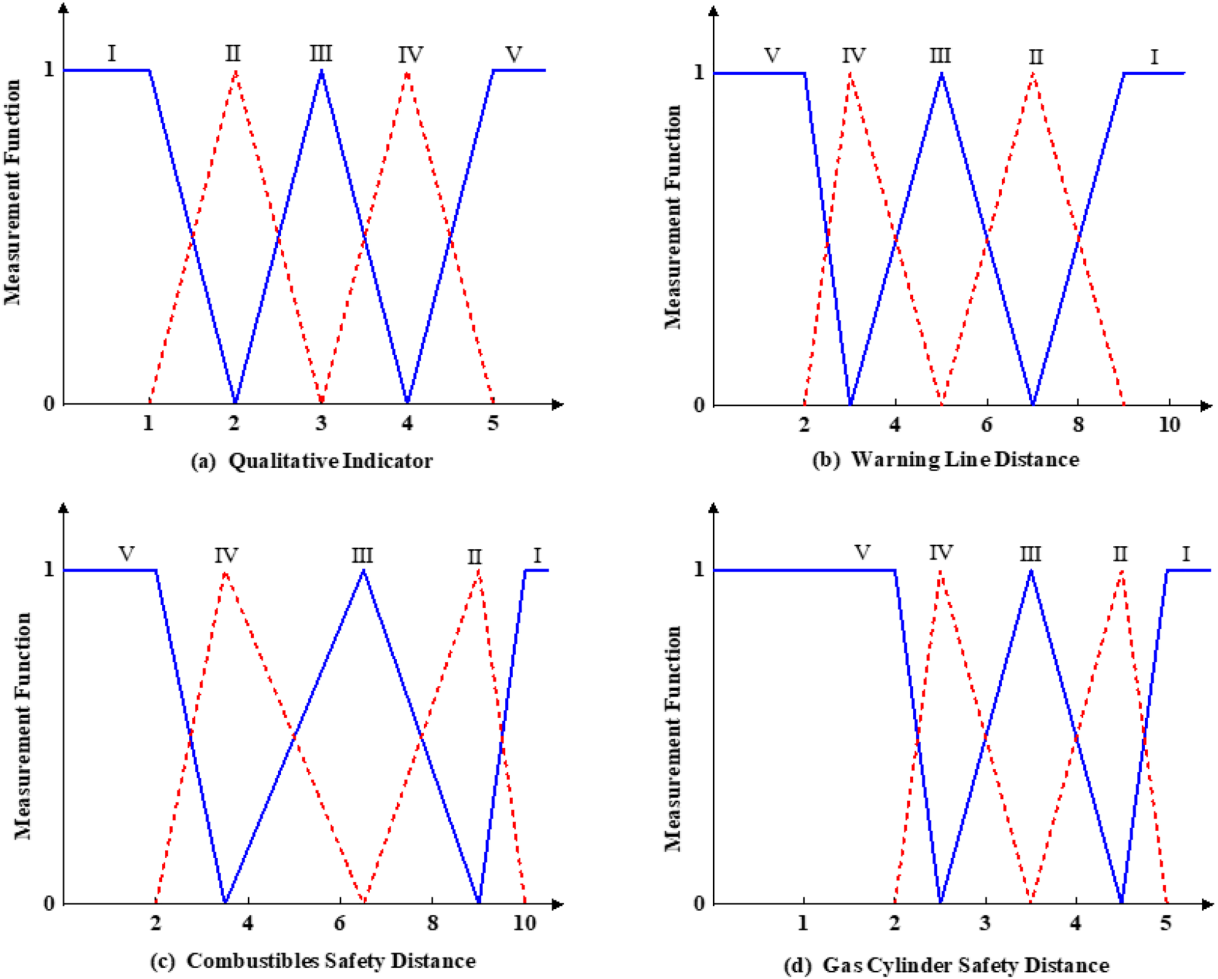
Single-indicator unascertained measurement function.

According to Table 2, = the risk evaluation indexes of hot work operation include qualitative indexes and quantitative indexes, and some qualitative indexes must be combined with expert scores and the actual mine conditions to obtain more accurate evaluation results. The values assigned to the risk indicator factors involved in the hot work operations process in the shaft (= #1) and tunnel (#2) are listed in Table 3. In the case of analysing hot work operations in the shaft, according to the definition of the single-indicator measurement function and assignment results of the evaluation indicator factors in Table 3, the single-indicator unascertained measure function is used to construct the unknown measurement function of each indicator for evaluating the risk of hot work operation in underground mines, as shown in Fig. 2. Among them, Fig. 2a shows a linear graph of the single factor measurement function for 16 qualitative evaluation indicators. The 16 qualitative indicators are pre-job safety training (X_1_), PPE usage (X_2_), proper operation (X_3_), licenced operation (X_4_), cylinder stabilization (X_7_), grounding of welding machine shell (X_8_), flame-resistant cables (X_9_), toxic and hazardous gas detection (X_10_), worksite lighting (X_12_), pumice on the work site slab (X_13_), hot work operations classification and procedures (X_14_), hot work permits (X_15_), risk factor identification (X_16_), specialized emergency protocols (X_17_), fire extinguishers (X_18_), and warning signs (X_19_). The single factor measurement functions for the 3 quantitative evaluation indicators are shown in Fig. 2b–d.

**Table 3:** Assignment of risk assessment indicators in mine shafts and tunnel.

Indicators	X_1_	X_2_	X_3_	X_4_	X_5_	X_6_	X_7_	X_8_	X_9_	X_10_
1^#^	3.5	3.8	4	5	5	6.5	5	4.3	4	3
2^#^	3.5	4	4.8	5	5.2	7	6.5	4.5	5	5

Indicators	X_11_	X_12_	X_13_	X_14_	X_15_	X_16_	X_17_	X_18_	X_19_	–
1^#^	12	3	5	1.75	1.6	2.4	1.8	3	2	–
2^#^	15	3.5	6	1.75	1.6	3	2	3	2.5

Based on the indicator assignments values in Table 3 and the above graphs of each single-indicator measurement function, the single-indicator measurement matrix ${\left({\upmu }_{1jk}\right)}_{19\times 5}$ for hot work operations in the shaft can be obtained as follows:

$${\left({\upmu }_{1jk}\right)}_{19\times 5}=\left[\begin{array}{ccccc}0& 0.5& 0.5& 0& 0\\ 0& 0.2& 0.8& 0& 0\\ 0& 1& 0& 0& 0\\ 1& 0& 0& 0& 0\\ 0& 0& 1& 0& 0\\ 1& 0& 0& 0& 0\\ 1& 0& 0& 0& 0\\ 0.3& 0.7& 0& 0& 0\\ 0& 1& 0& 0& 0\\ 0& 0& 1& 0& 0\\ 1& 0& 0& 0& 0\\ 0& 0& 1& 0& 0\\ 1& 0& 0& 0& 0\\ 0& 0& 0& 0.25& 0.75\\ 0& 0& 0& 0.4& 0.6\\ 0& 0& 0.6& 0.4& 0\\ 0& 0& 0& 0.2& 0.8\\ 0& 0& 1& 0& 0\\ 0& 0& 0& 1& 0\end{array}\right]$$

### Calculation of the combined weights of the evaluation indicators

#### Determination of the subjective weights

The order relationships and relative importance of the risk factors at the various levels of hot work operations were determined by multiple experts and management personnel in the field of metal mining. The findings are provided in Table 4.

**Table 4:** The order relationship and relative importance of indicators at different levels.

Indicator level	Order of importance	Order relationship	Relative importance(${r}_{k}$)
Human factors	X_3_ > X_1_ > X_4_ > X_2_	${X}_{1}^{*}{>X}_{2}^{*}>{X}_{3}^{*}>{X}_{4}^{*}$	1.2, 1.4, 1.2
Equipment factors	X_6_ > X_8_ > X_7_ > X_9_ > X_5_	${X}_{5}^{*}{>X}_{6}^{*}>{X}_{7}^{*}>{X}_{8}^{*}>{X}_{9}^{*}$	1, 1.2, 1.2, 1.4
Environmental factors	X_11_ > X_10_ > X_13_ > X_12_	${X}_{10}^{*}{>X}_{11}^{*}>{X}_{12}^{*}>{X}_{13}^{*}$	1.2, 1.4, 1.8
Management factors	X_16_ > X_14_ > X_15_ > X_17_ > X_18_ > X_19_	${X}_{14}^{*}{>X}_{15}^{*}>{X}_{16}^{*}>{X}_{17}^{*}>{X}_{18}^{*}>{X}_{19}^{*}$	1.2, 1, 1, 1.2, 1.4

Subjective weights for all indicators were calculated based on relative importance (${r}_{k}$) and Eq. (8). For example, regarding the human factor indicator hierarchy, ${r}_{2}={W}_{1}^{*}/{W}_{2}^{*}=1.2$, ${r}_{3}={W}_{2}^{*}/{W}_{3}^{*}=1.4$, and ${r}_{4}={W}_{3}^{*}/{W}_{4}^{*}=1.2$, the calculated weights ${W}^{*}$ of ${X}_{1}^{*}{, X}_{2}^{*}, {X}_{3}^{*}, {X}_{4}^{*}$, are ${W}^{*}$ = (0.3419, 0.2849, 0.2035, 0.1696), and the subjective weights of the indicators at the human factor level ${W}_{P}$ were obtained by replacing ${W}^{*}$ with the corresponding weights of the evaluation indicator set X, ${W}_{P}$  = (0.2849, 0.1696, 0.3419, 0.2035). The calculated subjective weights of the other hierarchical indicators were calculated as ${W}_{Eq}$ = (0.1233, 0.2485, 0.2071, 0.2485, 0.1726); ${ W}_{En}$ = (0.3020, 0.3624, 0.1198, 0.2157), and ${ W}_{M}\hspace{0.17em}=\hspace{0.17em}$(0.1776, 0.1776, 0.2132, 0.1776, 0.1481, 0.1058). The subjective weights of the primary indicators were (0.2835, 0.2835, 0.2362, 0.1969), and the calculation results for the subjective weights of the indicators are listed in Table 5.

**Table 5:** Subjective weight of evaluation indicators.

Indicators	X_1_	X_2_	X_3_	X_4_	X_5_	X_6_	X_7_	X_8_	X_9_	X_10_
Level	0.2849	0.1696	0.3419	0.2035	0.1233	0.2485	0.2071	0.2485	0.1726	0.3020
Weight	0.0808	0.0481	0.0969	0.0577	0.0350	0.0704	0.0587	0.0704	0.0489	0.0713

Indicators	X_11_	X_12_	X_13_	X_14_	X_15_	X_16_	X_17_	X_18_	X_19_	–
Level	0.3624	0.1198	0.2157	0.1776	0.1776	0.2132	0.1776	0.1481	0.1058	–
Weight	0.0856	0.0283	0.0509	0.0419	0.0419	0.0504	0.0419	0.0350	0.0250	–

#### Determination of the objective weights

Based on the single-indicator measurement matrix and Eqs. (2)–(5), the entropy value and objective weight of each indicator were calculated as listed in Table 6.

**Table 6:** Entropy and objective weight of evaluation indicators.

Indicators	X_1_	X_2_	X_3_	X_4_	X_5_	X_6_	X_7_	X_8_	X_9_	X_10_
Entropy	0.3051	0.2719	0.2236	0.1955	0.1869	0.1955	0.1955	0.3032	0.2236	0.1869
Weight	0.0488	0.0512	0.0545	0.0565	0.0571	0.0565	0.0565	0.0490	0.0545	0.0571

Indicators	X_11_	X_12_	X_13_	X_14_	X_15_	X_16_	X_17_	X_18_	X_19_	–
Entropy	0.1955	0.1869	0.1955	0.3740	0.4121	0.3352	0.3622	0.1869	0.2239	–
Weight	0.0565	0.0571	0.0565	0.0436	0.0413	0.0467	0.0448	0.0571	0.0545	–

#### Determination of the combined weights

Asshown in Tables 4 and 5, there is a certain difference between the subjective and objective weights. Therefor it is necessary to calculate the combination of weights of the dynamic hot work operation process by Eq. (10), and the results are provided in Table 7.

**Table 7:** Combination weight of hot work evaluation indicators.

Indicators	X_1_	X_2_	X_3_	X_4_	X_5_	X_6_	X_7_	X_8_	X_9_	X_10_
Weight	0.0325	0.0203	0.0436	0.0269	0.0165	0.0328	0.0274	0.0285	0.0220	0.0336

Indicators	X_11_	X_12_	X_13_	X_14_	X_15_	X_16_	X_17_	X_18_	X_19_	–
Weight	0.1690	0.0565	0.1006	0.0639	0.0605	0.0822	0.0657	0.0698	0.0476	–

### Determination of the multi-indicator uncertainty matrix and risk level

The evaluation vector of the comprehensive evaluation objects can be obtained by matrixing the combined weight vector of the hot work operation risk evaluation indicators and the single-indicator measurement matrix provided in Table 6 through Eq. (11) ${\upmu }_{ik1\#}$ = {0.3653, 0.1059, 0.2582, 0.1338, 0.1369}. The confidence level is $\lambda$ = 0.7 as calculated by the confidence identification criteria and Eq. (13), i.e., C1 + C2 + C3 = 0.7293 > 0.7. The safety risk level of tmine hot work operations was assessed as Class III, indicating a moderate risk level. Similarly, the calculated multi-index comprehensive measurement evaluation vector for hot work operations in tunnels is ${\upmu }_{ik2\#}$ = {0.3654, 0.1484, 0.1079, 0.1264, 0.2519}. According to the confidence identification criteria, C1 + C2 + C3 + C4 = 0.7481 > 0.7, indicates that the safety risk level of hot work operations in the mine tunnel is of Class IV and to the tunnel is classified as exhibiting a high risk.

### Result analysis

1. As described in section “Determination of the multi-indicator uncertainty matrix and risk level”, we can obtain a multi-indicator measurement matrix for the shaft and tunnel. According to the confidence identification criteria and Table 8, the risk level of hot work operation in the shaft is Level III, which belongs to the ‘moderate risk’ class, and the risk level of hot work operation in the tunnel of the mine is Level IIV, which belongs to ‘high risk’ class, which agree with the actual situation of the mine. The risk level of the 2 workplaces is 2# > 1#, i.e. the risk level of hot work operation is high in the tunnel than in the shaft, because the middle section of the mine extends from −390 to −630 m. This newly built system involves many pipeline welding, equipment maintenance and other projects, which increase the risk level based on the site conditions.
2. According to the results of the combination weights of the indicators in Table 7, combustible materials at the work site (X11) exhibit the highest weight (0.1690), indicating that the main cause of accidents due to fire operations is that combustible materials at the fire operation site are not removed, and high-temperature particles generated by impacting the surface of combustible materials trigger fire accidents. This is followed by the management of factors in the field of fire operations to identify the risk factors at the work site (X16), with a comprehensive weight of 0.0822. This value indicates that before fire operations, a detailed operation plan should be formulated for the operation site, and the risk and harmful factors at the site should be comprehensively recognized to ensure favourable safety preparation. Finally, the operation plan is an indicator of correct operations at the operation site in terms of personnel factors (X3), with a comprehensive weight of 0.0464, which indicates that compliant operation by personnel in the fire operation process exerts a greater impact on the whole fire operation project.
3. In terms of safety management, mines should develop a strict hot work operation approval system and operation site supervision system, from the intrinsic safety aspect to improve the hot work operation safety risk control, standardize the personnel operation behaviors, and realize effective control of whole stage hot work operation risk factor.

## Conclusion

In this study, a novel risk evaluation method for underground mine hot work operations was proposed. This method incorporats both subjective and objective weightings approach into the evaluation process and was subsequently applied. The following conclusions can be obtained:

1. The unascertained measurement theory is applied in risk analysis of hot work operations in noncoal mines, leading to the development of an unascertained measurement model for assessing the risk throughout the entire process of hot work operations. The utilization of the confidence identification criteria effectively determines the final risk level of hot work operations. This approach successfully addresses the issues of multifaceted, fuzzy, and uncertain factors in the evaluation of risk levels in noncoal mine hot work operations.
2. A risk assessment index system for hot work operations in noncoal mines is established, considering human, equipment, environmental, and management factors. The combination of the combined weight method, the order relationship method, and the entropy weight method is introduced to determine both the subjective and objective weights for each evaluation indicator. This integration enhances the rationality and accuracy of the evaluation results.
3. By applying the method proposed in this paper in risk assessment of hot work operations in vertical shafts and tunnels in a metal mine, we obtained risk level III (moderate Risk) for vertical shafts and risk level IV (high risk) for tunnels. Moreover, the risk assessment results for the two hot work operation areas showed a ranking of 1# > 2#. The evaluation process demonstrated high feasibility, and the results are consistent with the actual on-site conditions. These results provide valuable reference information for risk management and assessment of hot work operations in mining enterprises.
4. Considering the risk levels of various indicators calculated previously and the problems present in actual hot work operations. Hot work supervisors should pay close attention throughout the entire hot work process to the following aspects: Firstly, the approval process of the work permit before hot work operations, safety training, and on-site safety inspections, with a particular emphasis on the removal of combustibles on-site as a critical component; secondly, the monitoring of dangerous, toxic, and harmful gases during the hot work process; and lastly, the extinguishing of fire sources after the completion of hot work operations. Hot work is one of the significant risks for mine fires and explosions. Effectively isolating combustibles and dangerous flammable and explosive gases at the hot work site can significantly reduce the incidence of fire accidents caused by hot work operations.

The underground mine environment is complex. The limitation of this model is that the selected evaluation indicators cannot fully cover all potential risk factors, and indicators of human factors, such as the professional level, experience and behaviour. during hot work are difficult to quantify. And the model calculation process lacks a risk likelihood scale similar to that of literature^42–45^, which does not provide a specific and comprehensive risk measurement framework This model provides an important theoretical basis and assessment framework for risk management of underground mine hot work operations. Regarding other restricted space activities in mines only the evaluation indicators and indicator risk levels should be adjusted to obtain reasonable risk assessment results, which can provide a systematic reference for mining enterprises in safety management.

## Data Availability

The datasets generated during and/or analyzed during the current study are available from the corresponding author on reasonable request.
